# SMAC Mimetics as Therapeutic Agents in HIV Infection

**DOI:** 10.3389/fimmu.2021.780400

**Published:** 2021-11-26

**Authors:** Bengisu Molyer, Ashok Kumar, Jonathan B. Angel

**Affiliations:** ^1^ Chronic Disease Program, Ottawa Hospital Research Institute, Ottawa, ON, Canada; ^2^ Department of Biochemistry, Microbiology and Immunology, University of Ottawa, Ottawa, ON, Canada; ^3^ Apoptosis Research Center of Children’s Hospital of Eastern Ontario, Department of Microbiology and Immunology, University of Ottawa, Ottawa, Canada; ^4^ Division of Infectious Diseases, Ottawa Hospital, Ottawa, ON, Canada

**Keywords:** SMAC mimetics, HIV, HIV latency, apoptosis, HIV therapeutics

## Abstract

Although combination antiretroviral therapy is extremely effective in lowering HIV RNA to undetectable levels in the blood, HIV persists in latently infected CD4^+^ T-cells and persistently infected macrophages. In latently/persistently infected cells, HIV proteins have shown to affect the expression of proteins involved in the apoptosis pathway, notably the inhibitors of apoptosis proteins (IAPs), and thereby influence cell survival. IAPs, which are inhibited by endogenous second mitochondrial-derived activators of caspases (SMAC), can serve as targets for SMAC mimetics, synthetic compounds capable of inducing apoptosis. There is increasing evidence that SMAC mimetics can be used to reverse HIV latency and/or kill cells that are latently/persistently infected with HIV. Here, we review the current state of knowledge of SMAC mimetics as an approach to eliminate HIV infected cells and discuss the potential future use of SMAC mimetics as part of an HIV cure strategy.

## Introduction

Infection with Human Immunodeficiency Virus (HIV), if untreated, leads to profound immune dysfunction and the development of Acquired Immune Deficiency Syndrome (AIDS) (1). Although the introduction of combination antiretroviral therapy (cART) has dramatically decreased AIDS related morbidity and mortality, effective treatment requires lifelong adherence to therapy in order to maintain plasma viremia below levels of detection and preserve immune function (2). When cART is interrupted, rapid rebound in viremia occurs which is due to the activation of HIV within latently infected CD4^+^ T-cells (3–5) and persistently infected macrophages (6). To lower the burden of therapy for people living with HIV with the goal of developing a permanent cure, novel therapeutic approaches are essential.

### HIV Can Persist in Cellular Reservoirs Despite cART

Cells infected with HIV harbor the HIV genome even under cART. The HIV provirus can establish a latent infection, exist as a persistent infection, or can be replication defective (7). In latent infection, no or very low amount of transcripts are produced (8, 9) whereas in persistent infection, a low number of virions can be continually released (6). In the case of replication defective proviruses, some HIV proteins may be produced but the mutations/deletions accumulated in the proviral genome prevent the formation of a replication competent virus (10).

The two main targets for HIV are CD4^+^ T-cells and tissue resident macrophages. Memory CD4^+^ T-cells are the main reservoir for latent infection. Although the prevalence of latently infected CD4^+^ T-cells is extremely low (estimated to be 1 in 1x10^6^ CD4^+^ T-cells in cART treated patients) (5, 11) these cells remain a major barrier for finding an HIV cure.

Tissue resident macrophages are relatively resistant to the cytopathic effects of HIV (12) and support a persistent infection; HIV DNA, RNA, and p24 capsid protein can be readily detected in macrophages from people living with HIV on cART (13, 14). Moreover, tissue resident macrophages are present in immune privileged sites like the brain (15, 16) and the genital tract (13) and have a long half-life that can range from months for alveolar macrophages (17) to up to decades for microglial cells in the brain (15, 16).

### HIV Cure Strategies

Currently, multiple strategies are being investigated as possible approaches to cure HIV infection. Some of these focus on activating the latent reservoir followed by immune mediated elimination of the virus (shock and kill) (18), gene editing to excise/mutate the provirus (19) and using transcriptional gene silencing to preserve the mechanisms that lead to the maintenance of HIV latency to functionally prevent any HIV transcripts from forming (block and lock) (20). These and other cure strategies are thoroughly reviewed by David et al. (21) A therapeutic approach that has gained traction in the last few years is the use of SMAC mimetics, small pro-apoptotic proteins that mimic the endogenous second mitochondria-derived activators of caspases (SMAC) proteins, as either a component of a shock and kill strategy, or an approach to directly kill the latently/persistently infected cells.

## HIV and Apoptosis

### Apoptosis-an Overview

Apoptosis is one type of programmed cell death of which there are two main pathways: the extrinsic pathway, mediated by death receptors on the cell surface and the intrinsic pathway initiated by cellular stress.

The extrinsic pathway requires death receptors on the cell surface to engage with their death signal ligands. Well known signal pairs include tumor necrosis factor receptor-1 (TNFR1) and tumor necrosis factor (TNF), Fas receptor and Fas ligand (FasL), and tumor necrosis factor-related apoptosis-inducing ligand (TRAIL) and TRAIL Receptor-1 (DR4) and TRAIL Receptor-2 (DR5 (22). The Fas-FasL or TRAIL-DR4/5 interaction leads to the recruitment of the adaptor protein Fas-associated protein with death domain (FADD) and formation of death-inducing signaling complex (DISC), which cleaves procaspase-8 and -10 into active caspase-8/-10 (23, 24). Caspase-8/-10 then goes on to cleave and activate caspase-3/-7, resulting in cell death (25, 26). In the TNF signaling pathway, formation of complex 2a, a complex similar to DISC, is observed. This complex which consists of TNF and TNFR-1, TNF Receptor-Associated Death Domain (TRADD), receptor-interacting serine/threonine-protein kinase 1 (RIPK1), TNF receptor associated factor 2 (TRAF2), and Fas-associated protein with death domain (FADD), can activate caspase-8 to drive the cells down the apoptosis pathway (27, 28).

The intrinsic pathway, also known as the mitochondrial pathway, is initiated by cellular stress inducing factors such as DNA damage, growth factor deprivation, and endoplasmic reticulum stress. These stress signals lead to the formation of pores in the mitochondrial membrane, resulting in the release of cytochrome c and second mitochondrial-derived activators of caspases (SMAC) (29). SMAC are endogenous inhibitors of inhibitors of apoptosis proteins (IAPs). Their role in cell death and disease is discussed in more detail below. Following cytochrome c and SMAC release, the apoptosome is formed by cytochrome c creating a complex with apoptotic protease-activating factor 1 (Apaf1) and caspase 9 (30). This complex in turn recruits and activates the effector caspases 3 and 7, leading to cell death (31).

One protein family that is important in regulating apoptosis is the Bcl-2 family member proteins. These proteins play a role in regulating apoptosis at the mitochondria level. Some of these proteins, including Bcl-2, Bcl-X_L_ and Mcl-1 are anti-apoptotic, whereas others, namely Bad and Bax, are pro-apoptotic (32). The extrinsic and intrinsic apoptosis pathways converge when Bid, a Bcl-2 family member protein, gets cleaved into tBid by caspase 8 (33, 34). tBid translocates to the mitochondrial outer membrane, resulting in membrane permeabilization and activation of the intrinsic apoptosis pathway.

Another important protein family is the IAPs. IAP family members play a crucial role in regulating apoptosis by inhibiting caspases. IAPs carry a baculovirus IAP repeat (BIR) domain composed of N-terminal tandem repeat of about 70 amino acids (35). The three IAPs with primarily anti-apoptosis activity are X-linked inhibitor of apoptosis protein (XIAP), and cellular IAP1 and 2 (cIAP1 and cIAP2). These proteins also carry a C-terminal RING zinc finger domain which has ubiquitin (Ub) ligase (E3) activity and a Ub-associated domain (UBA) (36, 37).

XIAP can bind to the N-terminus of pro-caspase 9 *via* its BIR3 domain, effectively inhibiting procaspase-9 from self-dimerizing and becoming active (38). XIAP can also inhibit the executioner caspases-3 and-7 by binding to them *via* its BIR2 domain (39). cIAP1 and cIAP2 employ a different mechanism of inhibiting apoptosis. cIAP1 and cIAP2 can bind to TRAF2 *via* the BIR1 domain and ubiquitinate it with their E3 ubiquitin ligase activity, effectively blocking the formation of apoptotic Complex 2 and redirect the pathway to the canonical NFκB pathway favoring cell survival (40, 41). cIAP1 and cIAP2 also play a role in negatively regulating the non-canonical NFκB pathway. In the resting state, cIAP1 and cIAP2 ubiquitinate NIK, causing its degradation. However, when cIAP1 and cIAP2 are not present, NF-κB-inducing kinase (NIK) can accumulate in the cytoplasm to phosphorylate IKKα and drive the cell to non-canonical NFκB signaling (22).

### SMAC and SMAC Mimetics

SMAC, the best characterized antagonist to IAPs, is released from the mitochondria along with cytochrome c as a component of the intrinsic apoptosis pathway (42). The 55 amino acid long N-terminus of SMAC is cleaved when it is released from the mitochondria, exposing an Ala-Val-Pro-Ile (AVPI) motif which allows it to bind to IAPs such as XIAP, cIAP1 and cIAP2 (43). SMAC can dimerize and bind to the BIR2 and BIR3 domains of XIAP, inhibiting the interaction between XIAP and caspase 9 (44). SMAC can also bind the BIR3 domain of cIAP1 and cIAP2, leading to their autoubiquitination and subsequent degradation (45).

SMAC mimetics are synthetic small molecule peptides that mimic the N-terminal NH_2_-AVPI binding motif of SMAC proteins. SMAC mimetics are able to bind to the BIR domains of XIAP, cIAP1, and cIAP2, resulting in pro-apoptotic activity (46). Both endogenous SMAC and SMAC mimetics can successfully antagonize anti-apoptotic XIAP, cIAP1 and cIAP2 proteins, and are therefore pro-apoptotic (22). Since cIAP proteins are important regulators in the NFκB pathway, and NFκB is a regulator of TNFα expression, SMAC mimetics can enhance TNFα-dependent apoptosis (47, 48), TNFα-independent apoptosis (49), and TNFα-dependent necroptosis (50) in different disease models.

Both monovalent SMAC mimetics with one AVPI binding motif such as LCL-161 (Novartis) and Debio-1143 (Ascenta Therapeutics) and bivalent SMAC mimetics with two AVPI binding motifs such as AEG-40730 (Tocris Bioscience) and birinapant (Tetralogic Pharmaceuticals) have been developed for clinical use and are currently being investigated in several clinical conditions (51)

### Influence of HIV Proteins on Apoptosis

As is the case in autoimmune diseases, many cancers, and a number of infections, apoptosis pathways are dysregulated in HIV infection. Death of CD4^+^ T-cells infected by HIV is mainly due to apoptosisthus not surprisingly, various HIV proteins modulate apoptosis, the effects of which depend on the host cell type, as reviewed by Timilsina and Gaur (52).

HIV Tat has been shown to have both anti-apoptotic and pro-apoptotic effects *in vitro*. Jurkat cells and peripheral blood lymphocytes transfected with Tat protein have been shown to be significantly more resistant to the FasL mediated apoptosis compared to their untransfected counterparts. Furthermore, anti-apoptotic proteins such as cFLIP and Bcl-2 were upregulated, whereas caspase-10 was downregulated in CD4^+^ T cells transfected with Tat (53). In contrast, Tat has been shown to upregulate FLICE and caspase-8 in HIV infected CD4^+^ T-cells making them more susceptible to apoptosis (54). Moreover, the addition of extracellular HIV Tat to monocytes results in the secretion TRAIL, and the apoptosis of bystander CD4^+^ T cells (55).

Similar to Tat, HIV Vpr has also been shown to have both anti- and pro-apoptotic effects. Vpr upregulates survivin, a protein that increase the stability of XIAP against proteasomal degradation, which in turn increases resistance to mitochondrial dependent apoptosis (56). Vpr also downregulates the expression of Bad in CD4^+^ T-cells (52) and upregulates the expression of Bcl-2 in both CD4^+^ T-cells and macrophages (57). Conversely, Vpr causes the release of cytochrome c from the mitochondria by binding to Bax or Ant (adenine nucleotide translocase) and voltage dependent ion channels in the mitochondrial membrane (58). However, cIAP1 and cIAP2 have been shown to protect persistently infected macrophages from the pro-apoptotic effects of Vpr (59).

HIV envelope is another key protein that affects the regulation of apoptosis. HIV envelope has been shown to upregulate cIAP1, cIAP2 and XIAP in macrophages which results in a degree of resistance to TRAIL-mediated apoptosis (60). However, both membrane bound and soluble gp120 have been shown to bind the CD4 receptor, leading to apoptosis of both infected and uninfected CD4^+^ T-cells (61).

In the latently infected CD4^+^ T-cell lines ACH2 and CEM, XIAP expression is increased and the inhibition of XIAP sensitizes these cells to apoptosis (62). Similarly, survivin and its upstream regulator OX40 are also upregulated in HIV infected resting CD4^+^ T-cells, supporting long term survival (63). Furthermore, persistently infected macrophages have been shown to be more resistant to apoptosis compared to their uninfected counterparts, and this is attributed to an increased Bcl2/Bax ratio (64, 65) and viral restriction factors (66).

However, it is important to keep in mind that changes in anti-apoptotic proteins in cells that make up the HIV reservoir from infected persons have not been assessed and the expression of anti-apoptotic proteins studied in cell lines latently infected with HIV may differ from that of the latently infected cells from people living with HIV.

## SMAC Mimetics in the Treatment of Disease

### SMAC Mimetics and Cancer

Resistance to apoptosis is one of the hallmarks of cancer (67). As several different cancers, including pancreatic cancer (68) and liver cancer (69) show increased expression of IAPs, it is no surprise that SMAC mimetics are being studied as potential cancer therapeutics. SMAC mimetics have been used to induce TNFα-dependent apoptosis (47, 48, 70) or TNFα-independent necroptosis (50, 71) in a variety of tumor cell lines, as reviewed by Silke et al. (28). Furthermore, there are a variety of SMAC mimetics in clinical trials for cancer. LCL-161 has shown to be well tolerated and was associated with disease stabilization (72) in Phase 1 studies for solid advanced tumors (NCT01098838). Similarly, birinapant has shown to be well tolerated in phase 2 studies for refractory ovarian cancer (NCT01681368) and was also associated with stabilization of disease (73). Moreover, a phase 2 study of Debio-1143 against squamous cell carcinoma of the head and neck (NCT02022098) demonstrated improved locoregional control of tumor compared to placebo (74). While SMAC mimetics have been well tolerated in the treatment of patients with advanced cancer, the threshold for such toxicities might be much different in generally healthy individuals with well treated HIV infection and, therefore, appropriate caution must be used in the clinical development of these small molecules.

### SMAC Mimetics and Hepatitis B Virus Infection

SMAC mimetics are potential therapeutic agents for chronic viral infections as well. The SMAC mimetic birinapant has been shown to promote the preferential elimination of hepatocytes infected with Hepatitis B virus (HBV) *via* TNFα-mediated cell death (75). Moreover, the SMAC mimetic APG-1387 (Ascentage Pharma) also showed a similar result by degrading cIAPs to promote immune mediating clearance of HBV infected hepatocytes and is currently being studied in a phase 1 clinical trial (NCT03585322).

## SMAC Mimetics and HIV

As stated above, HIV interferes with the regulation of apoptosis in infected cells. Hence, SMAC mimetics represent an exciting therapeutic candidate for use as part of an HIV cure strategy. The effect of SMAC mimetics on infected cells depends on the type of SMAC mimetic used and the infection model employed. Broadly, the studies that have used SMAC mimetics as a potential therapeutic agent against HIV can be categorized as:

1. SMAC mimetics that induce latency reversal in HIV infected cells
2. SMAC mimetics that lead to apoptosis of HIV infected cells

### SMAC Mimetics Can Reverse HIV Latency *via* the Noncanonical NFκB Pathway

**Figure 1:**
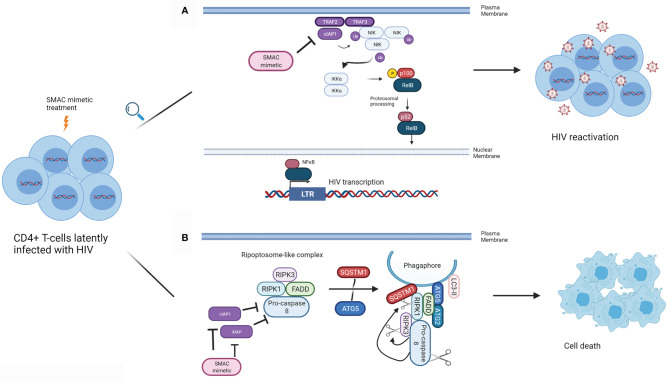
Mechanism of action of SMAC mimetics in latency reversal and apoptosis. **(A)** SMAC mimetics can induce latency reversal in HIV infected cells. TRAF2 and TRAF3 recruit NIK to cIAP1, and cIAP1 ubiquitinates NIK, resulting in its proteosomal degradation, preventing non-canonical NFκB signalling. SMAC mimetics bind to the BIR3 domain of cIAP1, which results in cIAP1’s autoubiquitination and degradation, freeing NIK. NIK accumulates in the cytoplasm, and activates IKKα, which in turn phosphorylates p100. p100 undergoes proteosomal processing to form p52, and the RelB/p52 NFκB complex translocates into the nucleus. NFκB can then bind to the LTR to inducing HIV gene expression leading to latency reversal. **(B)** SMAC mimetics can lead to apoptosis of HIV infected cells. SMAC mimetics cause the degradation of cIAP1 and XIAP, resulting in autophagy induction in HIV infected T_CM._ A ripoptosome-like DISC complex (Caspase 8, RIPK1, RIPK3, FADD), forms on unclosed phagophore membranes by using autophagy machinery (SQSTM1, ATG5, ATG2, LC3-II) as a scaffold, leading to selective killing of HIV infected CD4^+^ T_CM_. (Adapted from 63). (Created with Biorender.com).

cIAP1 represses the noncanonical NFκB pathway, and therefore has been presumed to contribute to the negative regulation of LTR-dependent HIV transcription (**Figure 1A**). Pache and colleagues have shown that this negative regulation can be overcome with the SMAC mimetic SBI-0637142 (76). Treatment with SBI-0637142 led to the degradation of cIAP1 in the latently HIV infected cell line J-lat 10.6 and resting CD4^+^ T-cells isolated from people living with HIV. As a result, a dose-dependent increase in HIV replication (GFP expression), with negligible cell death was observed in J-lat 10.6 cells treated with SBI-0637142. Furthermore, SBI-0637142 and the histone deacetylase inhibitor (HDACi) panobinostat work synergistically to reverse latency in J-lat 10.6 cells, as well as in two other J-lat derived latently infected cell lines, 2D10 and 5A8 (76). Moreover, the combination of SBI-0637142 and panobinostat was able to reverse latency in memory CD4^+^ T-cells from patients on cART, in a degree comparable to stimulation with CD3 and CD28 (76). The drug Ciapavir developed by the same group, which consists of a bivalent version of the SMAC mimetic SBI-0637142, has been shown to be more potent than SBI-0637142 alone in reversing HIV latency, both in cell lines such as J-lat 2D10 and in the humanized BLT mouse model of HIV. Ciapavir treatment resulted in an increase in HIV gag RNA in virally suppressed humanized BLT mice without triggering significant cytokine release or T-cell activation (77).

Similar to SBI-0637142, the SMAC mimetic Debio-1143 can degrade cIAP1 and lead to the activation of the non-canonical NFκB pathway and reverse latency in the latently infected cells lines J-lat 10.6, 2D10 and 5A8 and in resting CD4+ T cells isolated from cART treated patients (78). Moreover, the treatment of resting CD4^+^ T-cells from cART suppressed HIV infected humanized BLT mice with Debio-1143, led to the expression of HIV RNA, comparable to the levels seen with PMA and ionomycin stimulation (78).

AZD5582 is another attractive SMAC mimetic candidate. It has been shown to induce replication competent HIV from resting CD4^+^ T-cells from cART treated patients (79). Interestingly, AZD5582 has also been shown to successfully reverse latency, defined by increase in HIV in plasma, to more than 60 copies/mL, in cART suppressed SIV infected rhesus macaques 96 hours after the first dose without inducing global CD4^+^ T-cell activation in these animals (79).

The SMAC mimetic AZD5582 has also been used in combination with a bispecific CD3 and HIV envelope retargeting DART approach. Dual affinity targeting (DART) molecules are a bispecific antibody platform that can be used to bind two different targets at the same time (80). Dashti et al., have used DART molecules to retarget CD8^+^ T-cells to HIV infected cells that display the envelope protein on their surface as a potential therapeutic approach to reduce the viral reservoir in simian/human immunodeficiency virus (SHIV)-infected rhesus macaques. Since latently infected cells display a very low or non-existent amount of HIV envelope protein on their surface, a combination of AZD5582 and DART was used with the goal to first promote latency reversal with the SMAC mimetics and then kill the infected cells *via* DART in SHIV infected, cART treated macaques. Disappointingly, no significant increase in SHIV RNA levels in the plasma was observed and the combination treatment with AZD5582 and DART molecules did not result in a significant decrease in SHIV cell-associated DNA in peripheral blood, lymph node or bone marrow CD4^+^ T-cells.

### SMAC Mimetics Can Lead to Apoptosis of HIV Infected Cells

SMAC mimetics can potentially be used to kill HIV infected cells directly. Hattori et al., have shown that the SMAC mimetic birinapant can kill the latently HIV-infected U1 and ACH2 cell lines and that this effect can be enhanced if the birinapant treatment is used in combination with the PKC activator, PEP005 (81). The authors propose that treatment with PEP005 increases the transcription of TNFα, which in turn activates the canonical NFκB pathway. The addition of birinapant then shifts this pathway to that of caspase activation, resulting in cell death.

In resting central memory CD4^+^ T-cells (T_CM_) infected with HIV *in vitro*, cIAP1 and XIAP are upregulated (62, 82). Treatment of these cells with each of the SMAC mimetics embelin, birinapant or GDC-1052 led to the degradation of XIAP and cIAP1 and induced autophagy in HIV infected T_CM_ (82). Furthermore, each of these three SMAC mimetics was successful in inducing TNF-independent cell death in HIV infected T_CM_, while no such effect was observed in uninfected T_CM_. Moreover, treatment of T_CM_ isolated from HIV infected individuals undergoing cART with each of the SMAC mimetics mentioned above induced cell death. Interestingly, death of SMAC mimetic treated T_CM_ was not purely due to apoptosis but also dependent on autophagy machinery. This study suggests that the autophagy machinery might serve as a scaffold on which DISC forms to drive the HIV infected T_CM_ to the apoptosis pathway (82) (**Figure 1B**).

Studies in which SMAC mimetics are used as a potential therapeutic agent in HIV infection have not been limited to those of CD4^+^ T-cells. Following their previous study, Campbell et al., showed that XIAP and cIAP1 expression are significantly higher in *in vitro* HIV infected macrophages and each of the SMAC mimetics LCL-161, AT-406 and birinapant increased cell death in a dose dependent manner in these cells (49). Unlike in HIV infected T_CM_ from HIV-infected individuals, SMAC mimetic induced death in HIV infected Mϕ was TNF-dependent. Similar to HIV infected T_CM,_ however, SMAC mimetic-induced apoptosis of HIV infected Mϕ depended on autophagy machinery where this machinery most likely serves as a platform for DISC to assemble, linking autophagic and apoptotic pathways (49).

## Future Directions

Clearly, SMAC mimetics hold potential promise as components of future HIV cure strategies. SMAC mimetics can be used in “shock and kill” strategies to reverse latency, and therefore fulfill the “shock” component of this approach. They may also be able to directly eliminate latently HIV infected CD4^+^ T-cells and persistently HIV infected macrophages.

As shown by the studies outlined above, SMAC mimetics can also be used in combination with other agents, to improve their ability to kill target cells. For example, SMAC mimetics have been used with PKC activators to directly kill cells infected with HIV (81). Similarly, other anti-apoptotic proteins that are upregulated in latently HIV infected cells can be targeted. It has been shown that the combination of LRAs, HIV specific cytotoxic CD8^+^ T-cells and the Bcl-2 antagonist ABT-199 decreases the total amount of HIV DNA in resting CD4^+^ T-cells from PLWHIV (83). Considering the latency reversal aspect of SMAC mimetics, it is possible a similar synergy in cell death may be achieved when SMAC mimetics are combined with Bcl-2 inhibitors. Since most studies with SMAC mimetics have been done in cancer, it is possible to look to the cancer field to understand the interactions SMAC mimetics might have with other agents. For example, combining SMAC mimetics with monoclonal agonist antibodies against TRAIL receptors have been shown to cause RIP-1 dependent apoptosis in neuroblastoma cells (84). In HIV, studies have shown that treating lymphocytes from people living with HIV with recombinant TRAIL has reduced viral burden (85). Thus, combining SMAC mimetics with TRAIL agonists may be another approach to the killing of HIV infected cells. Another agent that can be combined with SMAC mimetics to create a similar effect is oncolytic viruses (OV). Notably, in the cancer field, the combination of SMAC mimetics and OV have been shown to act in synergy in eradicating tumors in a few different ways. Firstly, SMAC mimetics and OV in combination can sensitize tumor cells to TNFα-dependent killing. For example, using the engineered rhabdovirus VSVΔM51 expressing TNFα in combination with the SMAC mimetic LCL161 leads to durable cures in mice with syngeneic tumors (86). Secondly, the combination of SMAC mimetics and OV has been shown to synergize in creating a tumor microenvironment that enhances anticancer responses. A study by Kim et al., showed that infection with VSVΔM51 promotes T-cell recruitment while SMAC mimetic LCL161 treatment aids in rejuvenating exhausted tumor-infiltrating CD8^+^ T-cells, leading to CD8^+^ T-cell mediated tumor control in *in vivo* breast cancer models (87).

Both oncolytic virus and SMAC mimetic treatment separately have been shown to kill cells latently/persistently infected with HIV. Specifically, MG1, an oncolytic rhabdovirus closely related to VSV (88), has been shown to preferentially infect and kill latently HIV infected CD4^+^ T-cells (89) and macrophages persistently infected with HIV (90). It would be interesting to determine if there is a synergy between SMAC mimetics and MG1 or other OVs in killing HIV infected cells and controlling HIV infection *in vivo*.

## Conclusion

The key to most HIV cure strategies is eliminating cells that are latently/persistently infected with HIV. HIV infection alters the regulation of apoptosis in infected cells, thus making the use of SMAC mimetics a very attractive approach to selectively killing these cells. SMAC mimetics can be used directly to kill HIV infected cells, or they can be used in combination with other molecules to reverse latency, thus facilitating the elimination of infected cells. The promising results obtained in studies using SMAC mimetics demonstrate that this approach has the potential to become an important component of a future HIV cure strategy.

## Author Contributions

All authors listed have made a substantial, direct, and intellectual contribution to the work and approved it for publication.

## Conflict of Interest

The authors declare that the research was conducted in the absence of any commercial or financial relationships that could be construed as a potential conflict of interest.

## Publisher’s Note

All claims expressed in this article are solely those of the authors and do not necessarily represent those of their affiliated organizations, or those of the publisher, the editors and the reviewers. Any product that may be evaluated in this article, or claim that may be made by its manufacturer, is not guaranteed or endorsed by the publisher.
